# Bis(9)-(−)-Meptazinol, a novel dual-binding AChE inhibitor, rescues cognitive deficits and pathological changes in APP/PS1 transgenic mice

**DOI:** 10.1186/s40035-018-0126-8

**Published:** 2018-09-11

**Authors:** Yuhuan Shi, Wanying Huang, Yu Wang, Rui Zhang, Lina Hou, Jianrong Xu, Zhuibai Qiu, Qiong Xie, Hongzhuan Chen, Yongfang Zhang, Hao Wang

**Affiliations:** 1 Department of Pharmacology and Chemical Biology, Institute of Medical Sciences, Shanghai JiaoTong University School of Medicine, Shanghai, 200025 People’s Republic of China; 2 Department of Medicinal Chemistry, School of Pharmacy, Fudan University, Shanghai, People’s Republic of China

**Keywords:** Bis(9)-(−)-Meptazinol, AChE inhibitor, Alzheimer’s disease

## Abstract

**Background:**

Alzheimer’s disease (AD) is a progressive and irreversible neurodegenerative brain disorder, which is the most common form of dementia. Intensive efforts have been made to find effective and safe treatment against AD. Acetylcholinesterase inhibitors (AChEIs) have been widely used for the treatment of mild to moderate AD. In this study, we investigated the effect of Bis(9)-(−)-Meptazinol (B9M), a novel potential dual-binding acetylcholinesterase (AChE) inhibitor, on learning and memory abilities, as well as the underlying mechanism in the APP/PS1 mouse model of AD.

**Methods:**

B9M (0.1 μg/kg, 0.3 μg/kg, and 1 μg/kg) was administered by subcutaneous injection into eight-month-old APP/PS1 transgenic mice for four weeks. Morris water maze, nest-building and novel object recognition were used to examine learning and memory ability. Aβ levels and Aβ plaque were evaluated by ELISA and immunochemistry.

**Results:**

Our results showed that chronic treatment with B9M significantly improved the cognitive function of APP/PS1 transgenic mice in the Morris water maze test, nest-building test and novel object recognition test. Moreover, B9M improved cognitive deficits in APP/PS1 mice by a mechanism that may be associated with its inhibition of the AChE activity, Aβ plaque burden, levels of Aβ and the consequent activation of astrocytes and microglia in the brain of APP/PS1 transgenic mice. Most of important, the most effective dose of B9M in the present study is 1 μg/kg, which is one thousand of the dosage of Donepezil acted as the control treatment. Furthermore, B9M reduced Aβ plaque burden better than Donepezil.

**Conclusion:**

These results indicate that B9M appears to have potential as an effective AChE inhibitor for the treatment of AD with symptom-relieving and disease-modifying properties.

## Background

Alzheimer’s disease (AD) is a typical neurodegenerative brain disorder, which is the most common form of dementia. However, the molecular etiology of AD remains unclear [1]. The characteristic changes of AD in the brain are characterized by precipitated amyloid plaques (Aβ) [2], tau-protein aggregation [3], neuroinflammation [4], and decreased levels of acetylcholine (ACh) [5]. Multiple evidences have suggested that Aβ accumulation in the brain is the principal factor inducing other pathological features including the formation of neurofibrillary tangles (NFTs), the progressive loss or death of cholinergic neurons and the activation of immune system [6].

Acetylcholinesterase inhibitors (AChEIs), which ameliorate the cognitive and behavioral defects of the patients by enhancing central cholinergic neurotransmission, have been widely used for the treatment of mild to moderate AD [7]. However, high dosage of AChEIs could lead to side effects, such as gastrointestinal reactions, bradycardia and muscle spasm. And AChEIs can’t directly interact with Aβ to slow down or reverse the progression of AD. Therefore, the clinical effectiveness of AChEIs has still been questioned.

Given the complex and multifactorial etiology of AD, it is generally accepted that a multi-target therapeutic approach is very necessary for AD treatment [8]. Thus, multi-target directed-ligands (MTDLs) design has been proposed to be an advanced strategy to develop novel disease-modifying drugs for AD [9, 10]. It is therefore not surprising that Aβ, other than AChE, becomes a significant therapeutic target for the design of MTDLs to ameliorate symptoms and progression of AD simultaneously [11].

In recent years, a great number of studies have shown that the peripheral anionic site (PAS) of AChE greatly accelerates Aβ deposition and promotes the assembly of Aβ into fibrils [12, 13]. Blocking PAS is efficacious for the prevention of Aβ deposition by reducing insoluble Aβ and consequently facilitating Aβ clearance. Therefore, dual-binding AChEIs, which are able to bind to both the catalytic active site (CAS) and PAS simultaneously, are of particular interest in AD therapy. According to this strategy, Bis(9)-(−)-Meptazinol (B9M) was designed and synthesized by connecting two (−)-Meptazinols with nonamethylene by our group, in an effort to identify novel drug candidate for AD. Molecular docking has revealed that B9M bound to CAS and PAS via hydrophobic interactions with Trp86 and Trp286 of AChE respectively and two “water bridges” situated at the two wings of B9M stabilized this interaction [14]. In vitro studies showed that B9M could evidently inhibit AChE activity in a reversible and selective mode and prevent AChE-induced Aβ aggregation.

However, whether B9M could rescue cognitive impairment in the animal models of AD remains unknown. APP/PS1 transgenic mice, which overexpress the Swedish mutation of human amyloid precursor protein (APP) together with human presenilin-1 (PS1) deleted in exon 9, have shown cognitive deficits, Aβ deposits and cholinergic nerve degeneration mimicking AD pathology [15]. Therefore, in the present study, eight-month-old APP/PS1 mice were utilized to assess whether B9M could alleviate the learning and memory deficits and Aβ aggregation of AD with the aim of evaluating the potential of B9M for the treatment of AD.

## Methods

### Chemicals

B9M was synthesized by School of Pharmacy, Fudan University (Shanghai, China). Donepezil was from Sigma Aldrich (St Louis, MO, USA). Aβ monoclonal antibody (6E10) was purchased from Covance (Emeryville, CA, USA). Monoclonal antibody of glial fibrillary acidic protein (GFAP), an astrocyte-specific protein, was obtained from Millipore (Temecula, CA, USA). Polyclonal antibody of ionized calcium-binding adapter molecule 1 (IBA-1), a microglia-specific protein, was from Arigo biolaboratories (Taiwan, China). Amplex Red Acetylcholine/Acetylcholinesterase assay kit and Aβ_40_, Aβ_42_ ELISA kits were purchased from Invitrogen (Carlsbad, CA, USA). Pierce BCA protein assay kit was from Thermo Fisher Scientific (Rockford, IL, USA). All other reagents were obtained from commercial sources.

### Animals and treatments

APP/PS1 transgenic mice and their wild-type littermates were obtained from the Model Animal Research Center of Nanjing University. All the mice were housed in a temperature-controlled room (22–24 °C) with a 12 h light/dark circle, and allowed free access to food and water. Animals were treated in accordance with the Guide for the Care and Use of Laboratory Animal. The experiments were carried out under the approval of the Institutional Animal Care and Use Committee of Shanghai Jiaotong University School of Medicine.

**Fig. 1:**
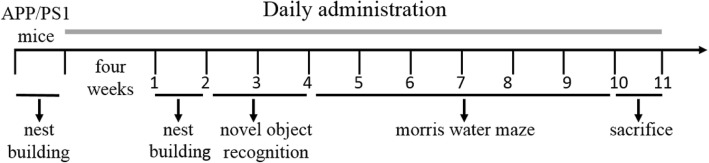
Scheme of experimental design. Bis(9)-(−)-Meptazinol (0.1 μg/kg, 0.3 μg/kg, 1 μg/kg), Donepezil (1000 μg/kg) or saline were administered to 8-month-old APP/PS1 mice for four weeks. Nest building was conducted before and after the administration following by the novel object recognition. Afterward, the Morris water maze was performed for six days. Then, the mice were sacrificed for histological and biochemical analyses

Eight-month-old APP/PS1 mice were randomly assigned into five groups (*n* = 9–11/group). Three B9M-treated groups were injected subcutaneously with B9M at the dose of 0.1, 0.3, 1 μg/kg into APP/PS1 mice for four weeks. Donepezil-treated group (1000 μg/kg) was administered by gavage. APP/PS1 and wild-type littermates mice were subcutaneously injected with equal volumes of 0.9% normal saline daily. After the treatment of four weeks, behavioral tests were carried out according to the experimental time schedule in Fig. 1.

### Behavioral tests

#### Morris water maze test (MWM)

The test was performed in a circular water tank (120 cm in diameter) containing opaque water (22 ± 1 °C) at a depth of 25 cm and dividing into four quadrants. A hidden escape platform (9 cm in diameter) was placed in the center of one quadrant, with its surface 1 cm below the water. The mice were subjected to acquisition trial four times a day for five consecutive days. During each trial, the mice were placed in water at one of the four starting positions and the starting position was randomly selected. The latency to reach the platform was measured using a computer-controlled video tracking system (Morris water maze video analysis system, Shanghai Yishu Software Technology Co., Shanghai, China). Each mouse was allowed to swim for 60 s. Mice that failed to find the hidden platform within 60 s were placed on it for 30 s. The same platform location was used for all mice. The platform was removed on the sixth day, and the mice were subjected to the spatial probe trial test for 60 s. The time and distance spent in the target quadrant were recorded.

### Nest-building test

Mouse was placed into an individual cage for 3 days. A nestlet (5 cm × 5 cm) was placed in the middle of the cage lined with fresh bedding 1 h before the night phase, ad libitum access to food and fresh water. No further environmental enrichment items were provided. After 24 h, the nests were assessed on a 5-point scale [16, 17]: 1 = less than 10% torn up, 2 = 10–50% torn up, 3 = less than 50% intact nestlet, 4 = recognizable but flat nest, 5 = a nearly doughnut-like nest.

### Novel object recognition test

The apparatus consisted of a square open field (30 cm × 30 cm × 30 cm). The objects used in the experiments were 50 ml plastic centrifuge tube (2.8 cm × 2.8 cm × 11.4 cm) and LEGO blocks (3 cm × 3 cm × 11 cm). All objects were of sufficient weight such that they could not be moved by the animals. The procedure of three 10-min trials was adopted from a previously publication with slight modification [18]. In the first trial (habituation), each mouse was placed individually into the empty chamber for 10 min. Twenty-four hours later, in the second trial (learning trial), two identical objects were placed into the open field 5 cm from the wall and let the mouse explore 10 min in the chamber. After 3 h (testing trial), one of the objects was replaced with a novel object and the mouse was put back into the chamber for 10 min. Objects and their placement in the open field were varied for each mouse to avoid positional biases. To control possible odor cues, the chamber and objects were cleaned with 75% ethanol at the end of each trial. Exploration was defined as direct contact of the nose or front paws with the object. The recognition index (RI) is defined as explorative time for the new object divided by the total explorative time for the both objects.

### Brain tissue preparation

After the behavioral tests, mice were randomly selected, anesthetized with 5% chloral hydrate and perfused transcardially with 0.9% saline. The brains of the mice were rapidly removed and immediately placed in 4% paraformaldehyde for fixation, embedded in paraffin, and then cut into sections (3 μm thick), which were placed at 4 °C for immunohistochemical analysis. Cerebral cortex and hippocampus of the remaining mice were harvested and dissected on an ice plate and then immediately stored in a − 80°Crefrigerator for further biochemical measurements.

### Immunohistochemistry examination

After being deparaffinized and rehydrated, the sections were treated with citric acid (pH 6.0) for 30 min followed by 20 min incubation with 0.3% peroxide. Subsequently, the sections were blocked with 10% normal horse serum and 1% BSA in TBS for 1 h, followed by incubation with primary antibodies overnight at 4 °C. The antigens of secondary antibodies were detected by standard ABC-DAB methods. Anti-6E10, anti-GFAP and anti-IBA1 antibodies were used to stain Aβ plaques, astrocytes, and microglia respectively. Then the slides were counterstained with hematoxylin and visualized using Leica Qwin software. Aβ plaques were also stained with 1% Thioflavin S (ThS) for 5 min in dark, and then washed with 70% ethanol once and distilled water twice (3 min each time). The images were captured using a fluorescence microscope and quantified with Image-Pro Plus 6.0 software.

### AChE activity assay

The cortex and hippocampus were homogenized in 9-fold (*w*/*v*) of 0.9% saline. The homogenate was centrifuged at 4000 g for 10 min at 4 °C. The supernatant was gathered for protein analysis. The BCA kit was used to quantify the concentration of extracted protein. The activity of AChE in cortex and hippocampus was determined using the Amplex Red Acetylcholine/Acetylcholinesterase assay kit. All procedures complied with the manufacturer’s instructions.

### Determination of Aβ levels

The cortex and hippocampus were homogenized with 3-fold (*w*/*v*) of RIPA lysis buffer, containing 1% proteinase inhibitor phenylmethylsulfonyl fluoride (PMSF). After incubation for 20 min on ice, the homogenized brain tissues were centrifuged at 14000  g for 1 h at 4 °C. The supernatant (soluble fraction) of brain lysates was collected to quantify soluble Aβ_40_ and Aβ_42_. The acquired pellet was incubated with guanidine buffer (6.25 M guanidine-HCl, 50 mM Tris-HCl, pH 8.0) containing 1% PMSF for 2 h at room temperature, and then centrifuged at 14000 g for 1 h at 4 °C [19]. The resultant supernatant was collected to quantify insoluble Aβ_40_ and Aβ_42._ The concentration of soluble Aβ and insoluble Aβ was determined using Aβ_40_ and Aβ_42_ ELISA kits according to the manufacturer’s instructions.

### Statistical analysis

Data were expressed as mean ± standard error of mean (SEM). The statistics were carried out by one-way or two-way analysis of variance (ANOVA) followed by the LSD test. The difference between two groups was assessed using Student’s t-tests. Statistical analysis was conducted by SPSS 13.0 software (Chicago, IL, USA) and *p* < 0.05 was considered statistically significant.

## Results

### Behavioral tests

#### B9M reversed the spatial learning and memory ability of APP/PS1 mice

**Fig. 2:**
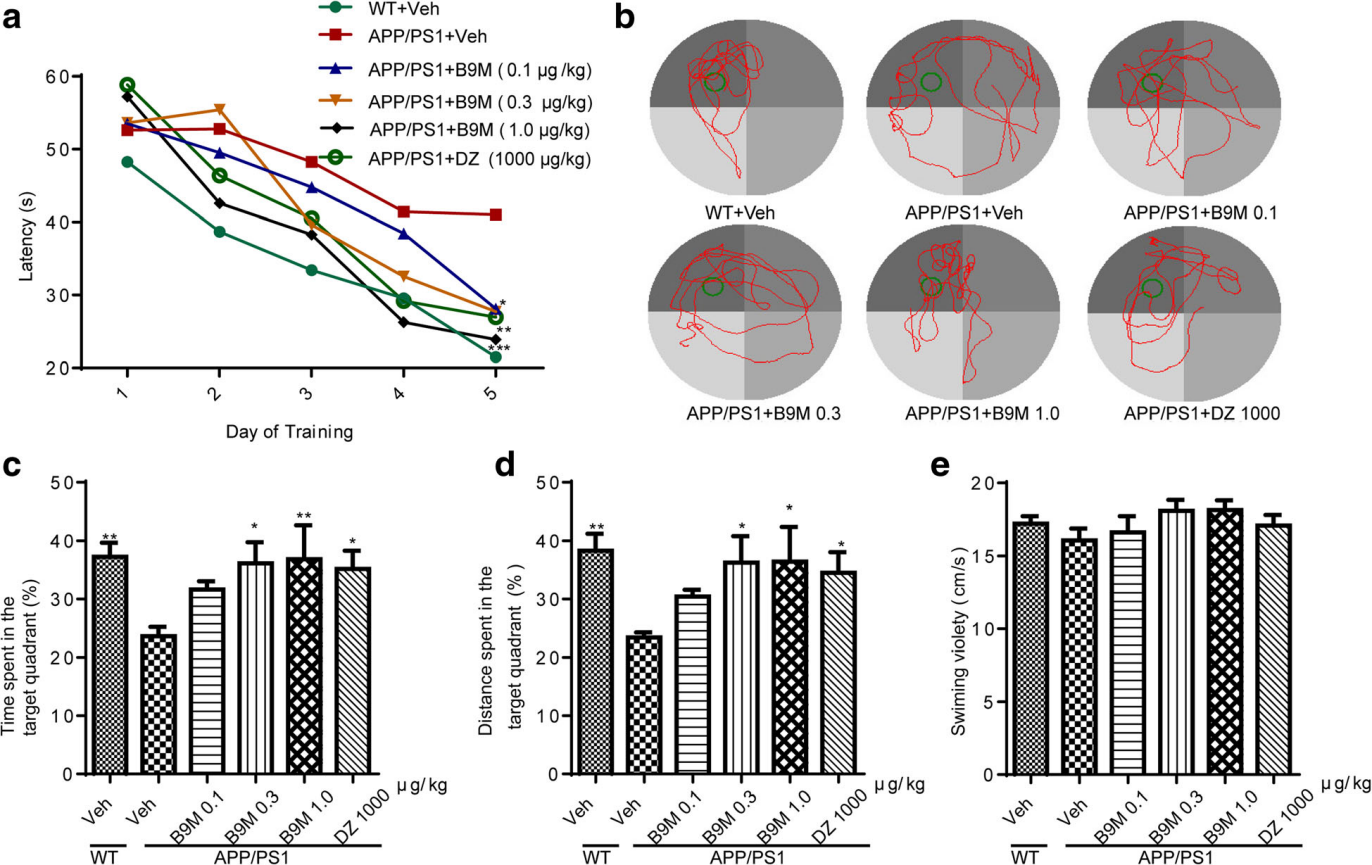
Bis(9)-(−)-Meptazinol ameliorated spatial learning and memory deficits of APP/PS1 mice in Morris water maze test. **a** Escape latency; **b** Representative path maps of each group; **c** Time percent in the target quadrant; **d** Distance percent in the target quadrant; **e** Swimming velocity. *N* = 9–11. Data represented as mean ± SEM, *p < 0.05, **p < 0.01, ***p < 0.001

To evaluate the spatial learning and memory ability of mice, Morris water maze test was performed including acquisition trial and probe trial, which primarily depends on the hippocampus. Wild type mice treated with vehicle and APP/PS1 mice treated with vehicle, B9M or Donepezil were assessed. The latency to the target platform for all groups during the 5 days of training is shown in Fig. 2a. As previously demonstrated, APP/PS1 mice exhibited a higher latency to locate the platform in the Morris water maze test than WT mice because of synaptic dysfunction and long-term potentiation (LTP) deficits in these animals [20]. Interestingly, we found that B9M administration for 4 weeks in 8 month-old APP/PS1 mice significantly reduced their latency to locate the platform, and the improvement in the spatial learning tasks occurred on fourth or fifth day (1 μg/kg on day 4: *p* < 0.05, 1 μg/kg on day 5: *p* < 0.01, vs APP/PS1 mice). On the sixth day, the platform in the pool was removed for the probe trail. As shown in Fig. 2c and d, the B9M-treated APP/PS1 mice stayed in the target quadrant for a longer time (0.3 μg/kg: *p* < 0.05, 1 μg/kg: *p* < 0.01, vs APP/PS1 mice), and swam shorter distance in the platform location (0.3 μg/kg, 1 μg/kg: *p* < 0.05, vs APP/PS1 mice). In addition, no significant difference was detected between Donepezil-treated APP/PS1 mice (1000 μg/kg) and B9M-treated APP/PS1 mice (1 μg/kg) (*p* > 0.05), indicating that B9M possessed higher potency than Donepezil. These findings were supported by representative images of routes of travel during the probe trail (Fig. 2b), which demonstrated that the mice treated with B9M swam closer to the previous hidden platform position than the APP/PS1 mice. No differences in swimming speed were present among all groups, which indicated that the observed differences in escape latencies and swimming time or distance were not due to the differences in locomotor ability (Fig. 2e).

#### B9M improved hippocampal-mediated nesting behavior in APP/PS1 mice

**Fig. 3:**
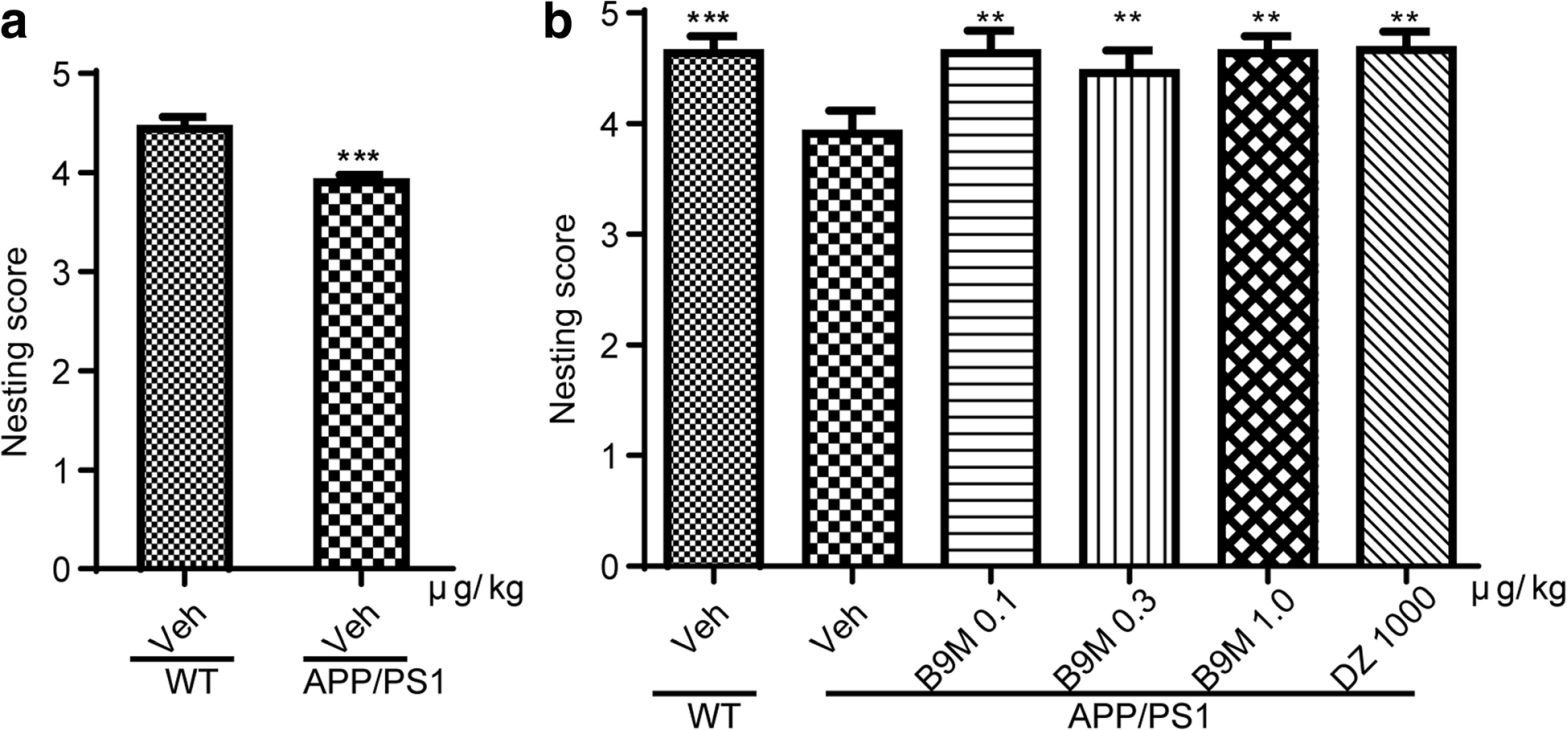
Bis(9)-(−)-Meptazinol improved the nesting score of APP/PS1 mice. (A) Nesting scores 24 h before treatment; (B) Nesting scores 24 h after four-week B9M treatment. N = 9–11. Data represented as mean ± SEM, **p < 0.01, ***p < 0.001

Nesting test, quantifying the ability of the mice to build a nest from a nestlet, was used as a reliable measure of hippocampal-mediated cognitive ability [21]. As shown in Fig. 3a, a significant decrease in nesting was observed in APP/PS1 mice before treatment (*p* < 0.001 vs wild type mice). Four weeks administration of B9M significantly reversed the nesting deficiency of APP/PS1 mice (0.1 μg/kg, 0.3 μg/kg, 1 μg/kg: *p* < 0.01, vs APP/PS1 mice) (Fig. 3b). And no significant difference was observed between the treatment with B9M at the dose of 1 μg/kg and that with Donepezil at the dose of 1000 μg/kg (*p* > 0.05), suggesting that the dosage of B9M to improve cognitive deficits is much less compared with Donepezil.

#### B9M enhanced novel object recognition in APP/PS1 mice

**Fig. 4:**
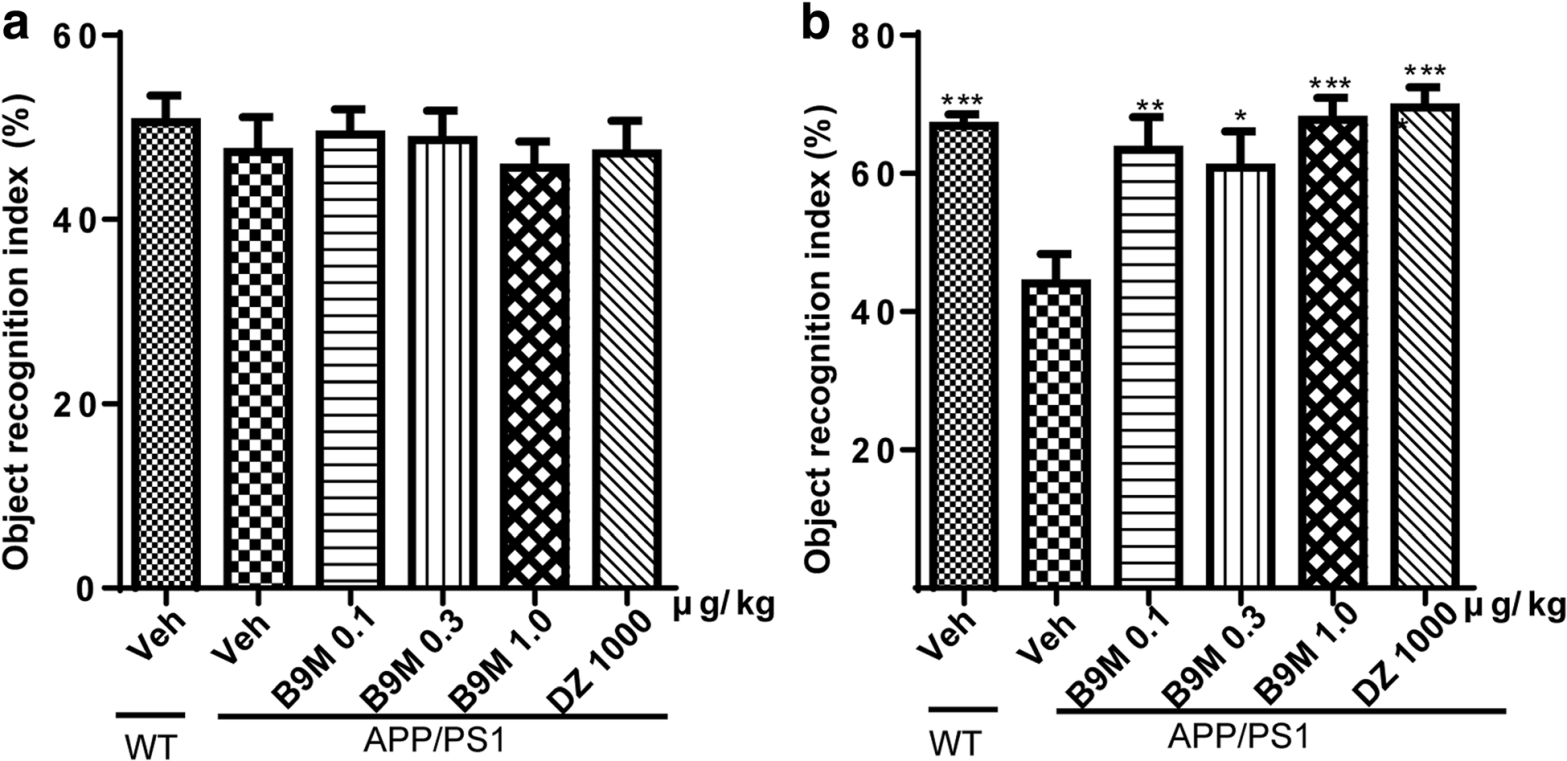
Bis(9)-(−)-Meptazinol reversed the novel object recognition deficits of APP/PS1 mice. Recognition index in mice during (**a**) learning phase; **b** testing phase. N = 9–11. Data represented as mean ± SEM, *p < 0.05, **p < 0.01, ***p < 0.001

The advantages of novel object recognition task are that there is no explicit need for food or water restriction and several behavioral endpoints can be rapidly obtained, including general activity, reactivity to novelty, and learning and memory [22]. As shown in Fig. 4a, after four weeks treatment of B9M, no significant differences were found in the time exploring two identical objects during the learning phase, which indicated that the mice had no preference for the position of the objects and surroundings. Three hours later, in the testing phase with two different objects (one novel, the other familiar), the recognition index (RI) had significant difference between APP/PS1 mice and wild type mice, suggesting that APP/PS1 mice failed to discriminate between novel and familiar objects (Fig. 4b). However, drug-treated groups preferred to explore the novel object. Compared with vehicle-treated APP/PS1 mice, 1 μg/kg B9M dramatically increased the RIs from 45.62 to 67.74% (*p* < 0.001), and 1000 μg/kg Donepezil increased the RIs from 47.15 to 70.46% (*p* < 0.001). These results suggest that B9M has an advantage in dosage than Donepezil enhance novel object recognition in APP/PS1 mice.

#### B9M inhibited AChE activity in APP/PS1 mice

**Fig. 5:**
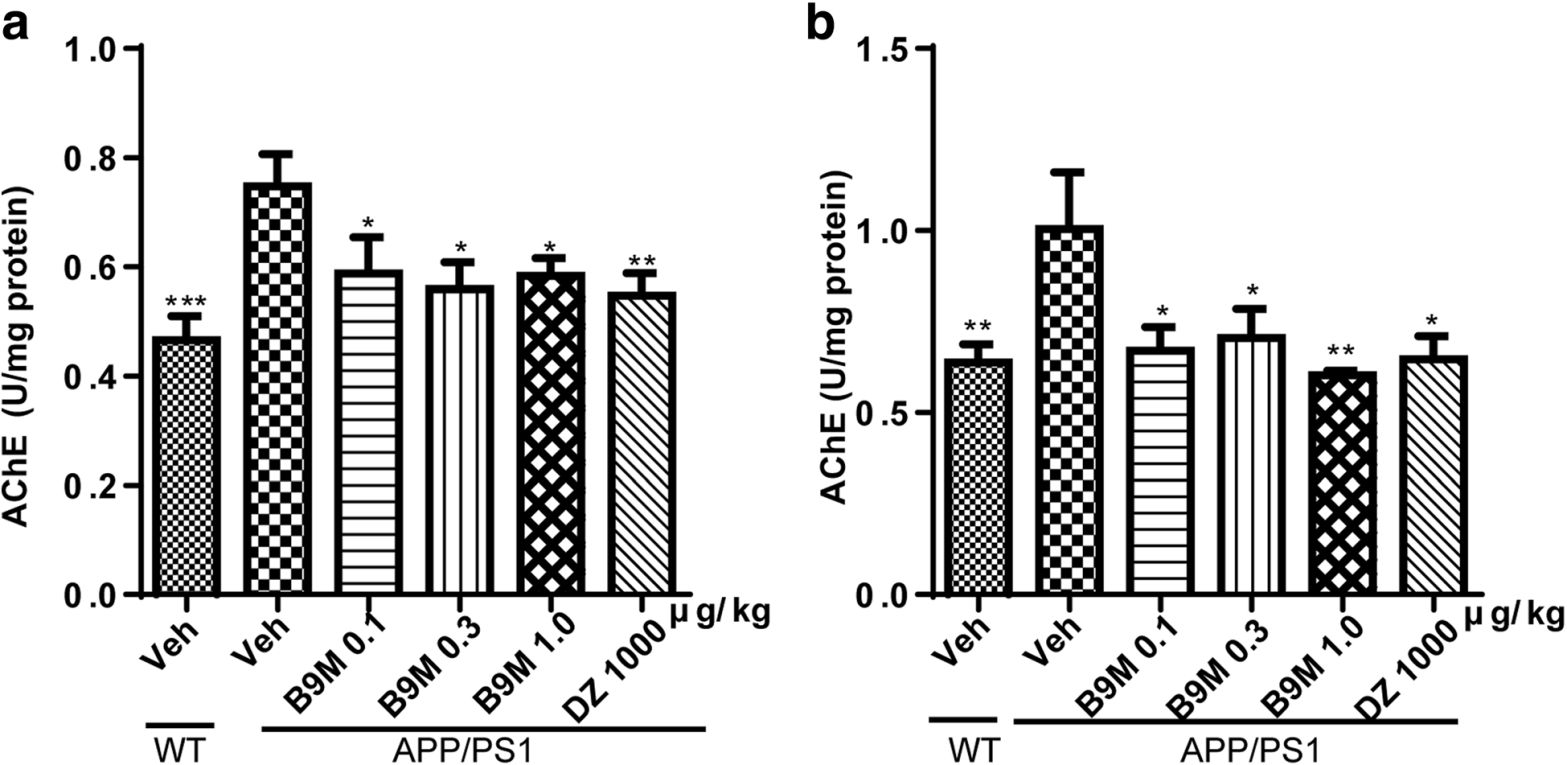
Bis(9)-(−)-Meptazinol inhibited AChE activity in APP/PS1 mice. **a** Cortex; **b** Hippocampus. *N* = 6–8. Data represented as mean ± SEM; *p < 0.05, **p < 0.01

It has been proved that cholinergic deficits generally lead to the memory and cognitive impairment in AD. It is generally known that AChE is the primary enzyme degrading ACh [23]. As shown in Fig. 5a and b, consistent with previous study, we found that AChE activity in the cortex and hippocampus of APP/PS1 mice was significantly elevated compared with wild type mice (*p* < 0.001 and *p* < 0.01 respectively). And compared with APP/PS1 mice, B9M administration for 4 weeks significantly reduced AChE activity in the cortex (0.1 μg/kg, 0.3 μg/kg, 1 μg/kg: *p* < 0.05, vs APP/PS1 mice) and hippocampus (0.1 μg/kg, 0.3 μg/kg: *p* < 0.05, 1 μg/kg: *p* < 0.01, vs APP/PS1 mice), which showed an obvious inhibitory effect of B9M on AChE activity. Most of all, the AChE activity in B9M-treated APP/PS1 mice at the dose of 1 μg/kg was remarkably inhibited to 77.80% in cortex (*p* < 0.05) and 60.06% in hippocampus (*p* < 0.01), similar to the inhibitory effect of Donepezil at the dose of 1000 μg/kg (*p* > 0.05), indicating that B9M could reverse the increased AChE activity in APP/PS1 mice.

#### B9M decreased Aβ in APP/PS1 mice

**Fig. 6:**
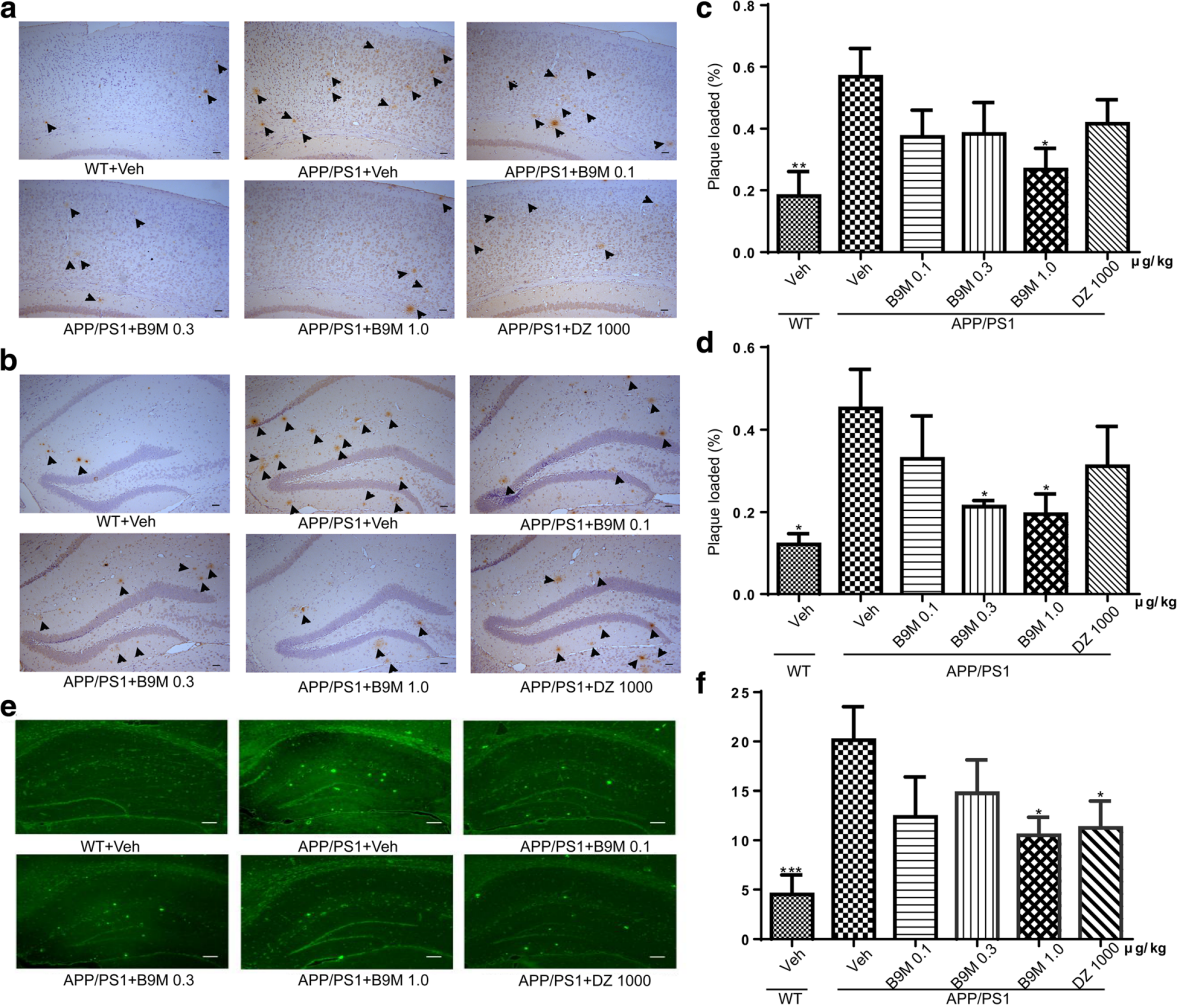
Bis(9)-(−)-Meptazinol decreased the amyloid plaques in APP/PS1 mice. **a**, **c** Immunohistochemical staining of 6E10-positive Aβ in cortex; **b**, **d** Immunohistochemical staining of 6E10-positive Aβ in hippocampus; **e**, **f** Thioflavin S-positive Aβ plaques in hippocampus. Scale bar = 50 μm in Fig. 6A and B, Scale bar = 100 μm in Fig. 6E, N = 4. Data represented as mean ± SEM, *p < 0.05, **p < 0.01, ***p < 0.001

To determine whether B9M could alter Aβ levels in the brain of APP/PS1 mice, the immunohistological and biochemical assays were performed. As shown in Fig. 6a and b, the nine-month-old APP/PS1 mice displayed strong Aβ immunoreactivity, a neuropathological manifestation of AD, compared with the wild type mice. Statistic results showed that the Aβ deposition in the B9M-treated mice was significantly reduced in the cortex (1 μg/kg: *p* < 0.05, vs APP/PS1 mice) and hippocampus (1 μg/kg: *p* < 0.05, vs APP/PS1 mice) compared with APP/PS1 mice, confirming the robustness of these results (Fig. 6c and d). In the meantime, Donepezil at the dose of 1000 μg/kg also inhibited Aβ deposition in APP/PS1 mice, but there was no significant difference compared with model group. In addition, these findings were clearly supported by an obvious decrease in the levels of the thioflavin S-positive senile plaques in the group treated with B9M at the dose of 1 μg/kg, which were significantly lower than those in the vehicle-treated APP/PS1 mice (*p* < 0.05) (Fig. 6e and f).

**Fig. 7:**
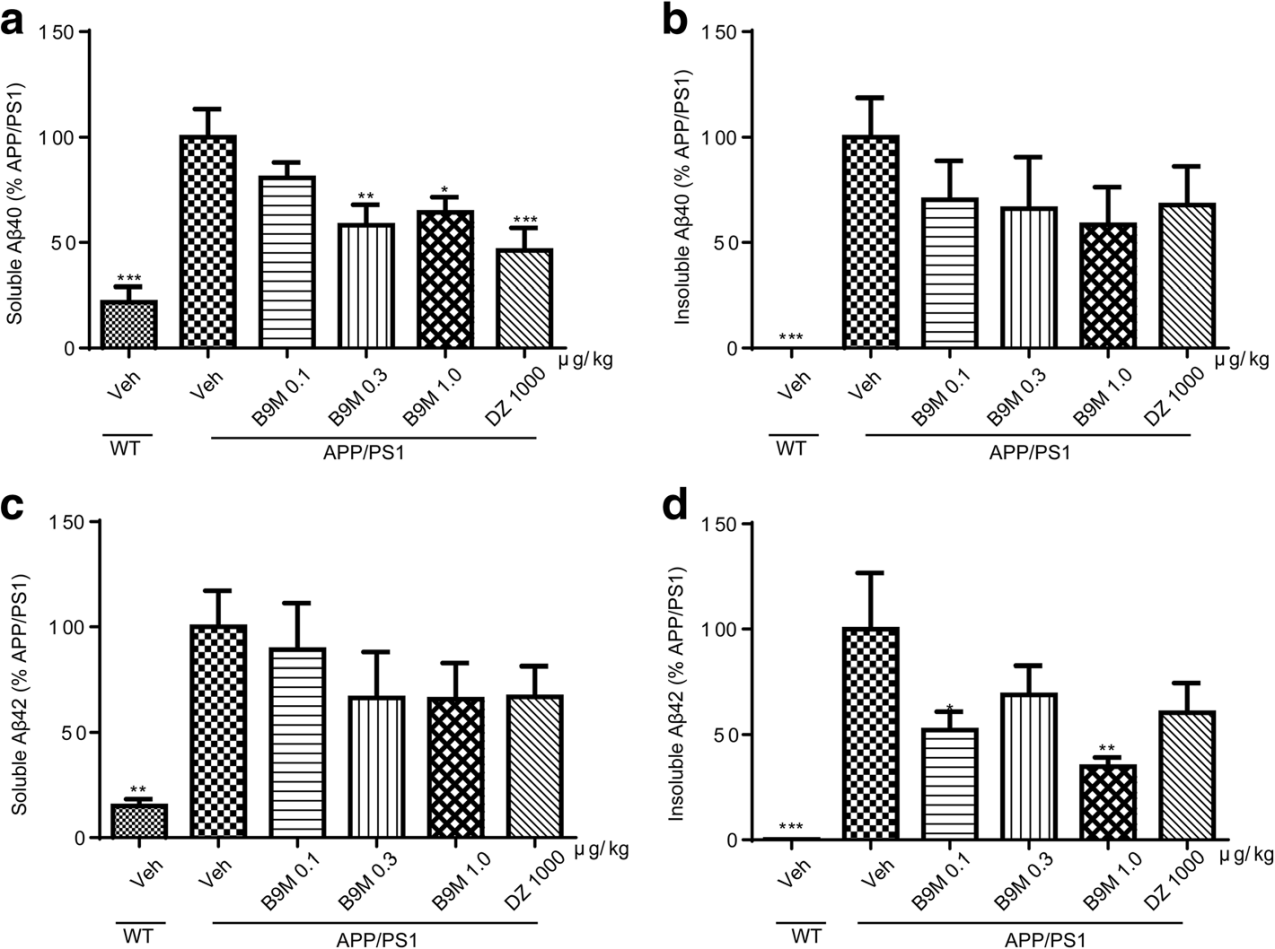
Bis(9)-(−)-Meptazinol treatment reduced Aβ levels in the cortex of APP/PS1 mice. **a** Soluble Aβ_40_; **b** Insoluble Aβ_40_; **c** Soluble Aβ_42_; **d** Insoluble Aβ_42_. N = 6–8. Data represented as mean ± SEM, *p < 0.05, **p < 0.01, ***p < 0.001

**Fig. 8:**
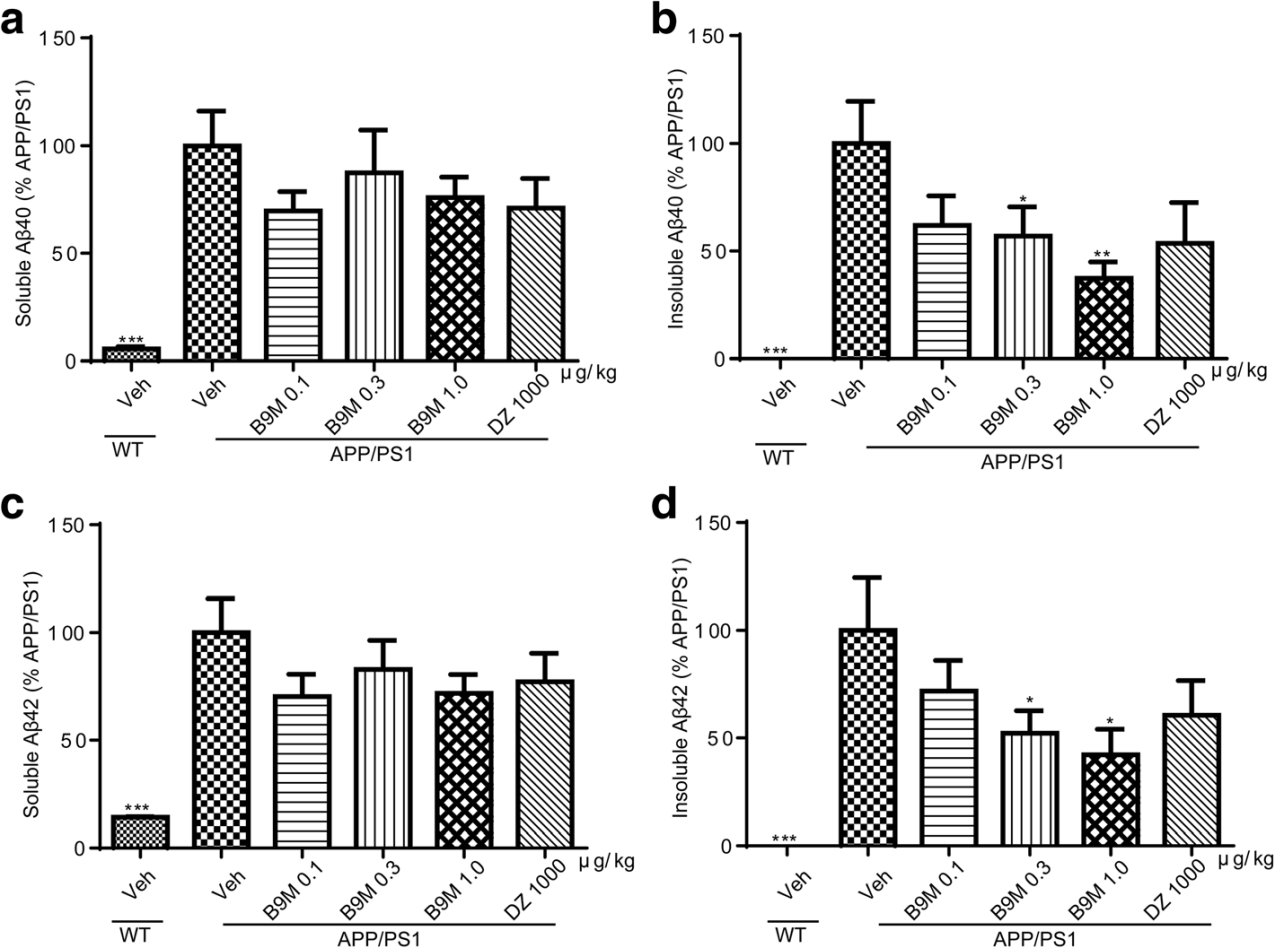
Bis(9)-(−)-Meptazinol treatment reduced Aβ levels in the hippocampus of APP/PS1 mice. **a** Soluble Aβ_40_; **b** Insoluble Aβ_40_; **c** Soluble Aβ_42_; **d** Insoluble Aβ_42_. N = 6–8. Data represented as mean ± SEM, *p < 0.05, **p < 0.01, ***p < 0.001

High levels of soluble and insoluble Aβ_40_ and Aβ_42_ were detected in the cortex and hippocampus of APP/PS1 mice by Sandwich ELISA assays, confirming our above observation. We detected a remarkable reduction in the soluble Aβ_40_ (0.3 μg/kg: *p* < 0.01, 1 μg/kg: *p* < 0.05, vs APP/PS1 mice) and insoluble Aβ_42_ (0.1 μg/kg: *p* < 0.05, 1 μg/kg: *p* < 0.01, vs APP/PS1 mice) levels in the cortex of B9M-treated APP/PS1 mice (Fig. 7a and d). Simultaneously, the insoluble Aβ_40_ and insoluble Aβ_42_ levels of the hippocampus with the treatment of B9M were significantly decreased in a dose-dependent manner (Fig. 8b and d). Together, these data showed that Aβ burden was significantly decreased by the administration of B9M in APP/PS1 mice.

#### B9M reduced the activation of astrocytes and microglia in APP/PS1 mice

**Fig. 9:**
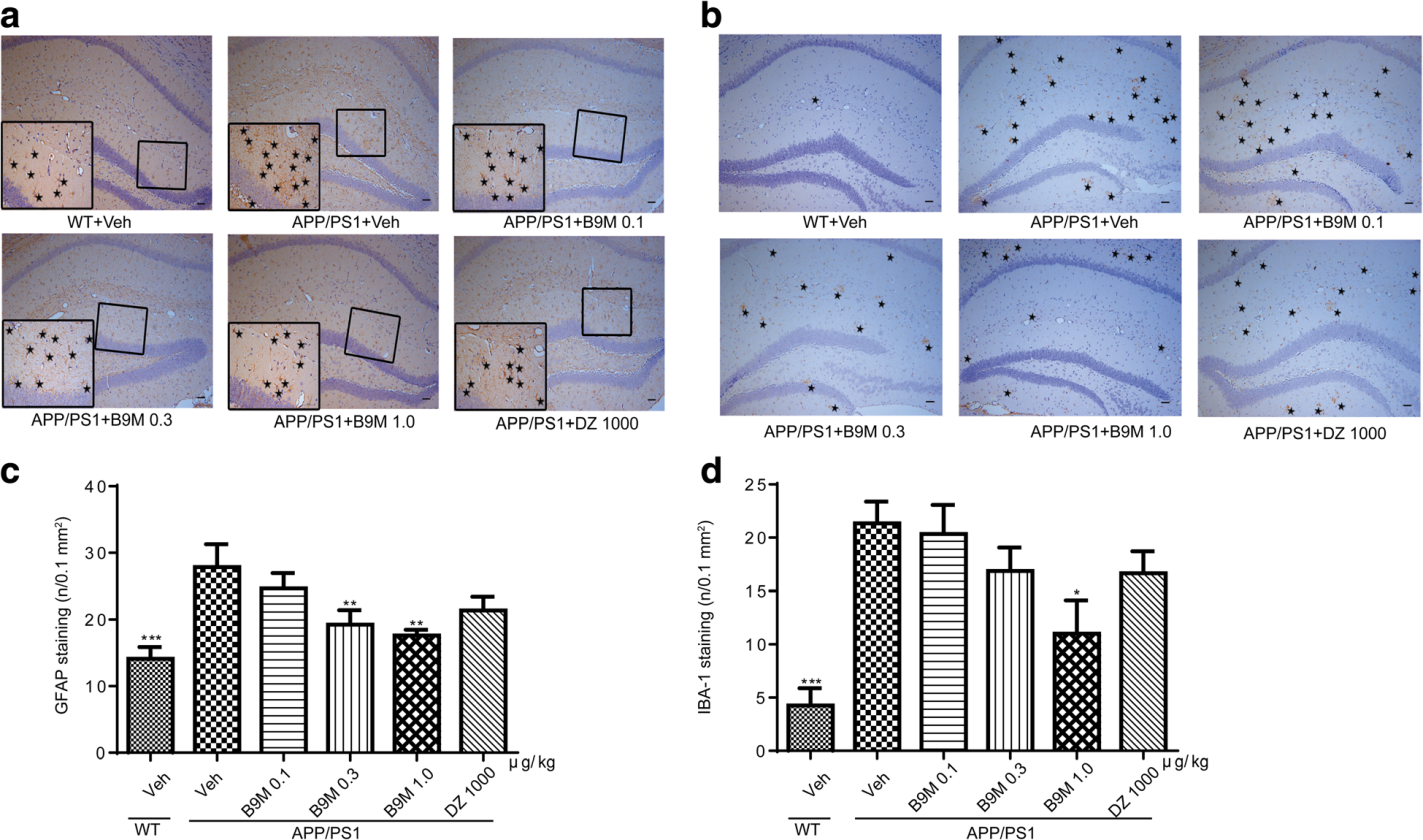
Bis(9)-(−)-Meptazinol reduced activated glial cells in APP/PS1 mice. **a**, **c** Glial fibrillary acidic protein (GFAP)-positive astrocytes in the hippocampus; **b**, **d** IBA1-positive microglia in the hippocampus. Scale bar = 50 μm, *N* = 4. Data represented as mean ± SEM,*p < 0.05, **p < 0.01, ***p < 0.001

Astrocyte activation is characterized by the appearance of a hypertrophic soma and processes and is often accompanied by an increase in the expression of GFAP, a major intermediate filament protein specific to astrocytes [24]. In the meantime, microglial activation is associated with the distribution of Aβ plaques and neurofibrillary tangles, which has been related to neurodegeneration, dementia progression and AD severity [25]. To identify whether B9M has an inhibitory effect on the activation of astrocytes and microglia cells, we evaluated GFAP and IBA1 immunoreactivity respectively (Fig. 9a and b). Consistent with previous results [26], our data revealed a significant increase in GFAP staining in APP/PS1 mice in contrast to wild type mice (Fig. 9c). However, B9M treatment decreased GFAP immunoreactivity in hippocampal in a dose-dependent manner (0.3 μg/kg: *p* < 0.01, 1 μg/kg: *p* < 0.01, vs APP/PS1 mice). In addition, we found that IBA1-positive microglia cells were significantly increased in APP/PS1 mice (*p* < 0.0001 vs wild type mice), which was remarkably decreased by B9M treatment (1 μg/kg, *p* < 0.05, vs APP/PS1 mice) (Fig. 9d). These results indicated that B9M evidently inhibited the activation of astrocytes and microglia in APP/PS1 mice.

## Discussion

Our study provided a property of B9M as a therapeutic compound that ameliorated the histopathological hallmarks of AD and reversed the associated cognitive and learning deficits in APP/PS1 mice. B9M treatment correlated to alleviating brain levels of Aβ as well as the soluble or insoluble Aβ_40_/Aβ_42_, inhibited the AChE activation and ameliorated astroglial and microglial reactivity in the hippocampus. In addition, treatment of APP/PS1 mice with B9M effectively improved cognitive ability and memory dysfunction compared to treatment of these mice with vehicle. Above all, 1 μg/kg is the optimal concentration to improve the learning and memory function and reverse the process of AD. Furthermore, the capability of B9M directly decreasing Aβ aggregation is stronger than that of Donepezil at the dose of 1000 μg/kg. The results indicate that B9M possesses a promising therapeutic effect in AD by improving the symptoms as well as modifying the disease.

Our previous studies showed B9M banded simultaneously on the CAS and PAS of AChE and exhibited high potent AChE inhibitory activity (IC_50_ = 3.9 nM). B9M also inhibited AChE induced Aβ aggregation, indicating its potential for AD treatment [14]. Furthermore, B9M showed memory ameliorating effects at the dose of 1 μg/kg in scopolamine-induced mice model [27]. Thus, based on our previous results, three doses of B9M (0.1, 0.3, 1 μg/kg) were administrated to APP/PS1 mice and three behavioral tests were conducted to evaluate learning and memory ability in the present study. Interestingly, the results of Morris water maze suggested that B9M influenced the time and distance percent in the target quadrant in a dose-dependent manner and 1 μg/kg was the optimal dose with the effect in accord with 1000 μg/kg Donepezil. Three doses of B9M showed similar cognitive improvement in novel object test and nest-building test possibly due to the different sensitivity of the behavior tests which are regulated by different regions of brain. For example, medial preoptic area and hippocampus are different to the treatment of B9M [28]. Therefore, it can be inferred that 1 μg/kg B9M could strongly rescue learning and memory deficits of AD mice, comparable with the effect of control drug Donpezil at the dose of 1000 μg/kg.

Our previous studies in vitro have confirmed that B9M, acted as dual-binding AChEI, not only had a high affinity with CAS to inhibit AChE activity, but also exerted preferential affinity with PAS, which led to strong inhibitory effect on AChE and AChE-induced Aβ accumulation. Thus, we tested the AChE activity as well as the accumulation of Aβ in these transgenic mice. Our findings suggested that B9M effectively decreased the AChE activity in cortex and hippocampus of APP/PS1 mice, which is consistent with the aforementioned studies [16]. Moreover, 1 μg/kg B9M and the 1000 μg/kg Donepezil have similar inhibitory effects on AChE activity.

Histopathological and biochemical analysis were applied to further explore the effect of B9M on plaque burdens and Aβ levels. The Aβ deposition was markedly decreased in B9M-treated group at the dose of 1 μg/kg compared with vehicle-treated transgenic mice. Meanwhile, B9M significantly reduced soluble and insoluble Aβ levels, which was confirmed by ELISA analysis. The optimum dose of B9M (1 μg/kg) was much lower than that of Donepezil (1000 μg/kg). Thus, it can be inferred that B9M directly or indirectly leads to fewer Aβ plaques via the combination with CAS and PAS of AChE.

The most commonly used anti-AD drug at present is AChEI, but it does not halt the pathological progression such as Aβ deposition during the course of the disease. Over the last two decades, the immunotherapies against Aβ were developed to treat AD. However, phase III trials of several anti-Aβ monoclonal antibodies failed to improve cognitive function in patients. Wang et al. demonstrated that the agent was not effective in removing Aβ plaques due to the saturation of antibody with soluble Aβ [29]. Our results showed that dual-binding AChEI B9M could inhibit AChE and reduce Aβ deposits, and might be better for late stage AD owning to the sustained symptomatic improvement and Aβ plaques reduction.

In addition, it has been found that the proliferation of microglia and astrocytes exists around Aβ deposition in the brain of AD model mice [24, 30] and in AD human brains [31]. Therefore, in order to clarify the effect of B9M on Aβ deposition-induced glial activation in APP/PS1 mice, we performed immunohistochemical staining of brain tissue with microglia and astrocyte-specific antibodies. Our results indicated that microglia and astrocyte activation reduced following B9M treatment at the dose of 1 μg/kg.

## Conclusion

In summary, this paper offers essential preclinical evidences that dual-binding site AChE inhibitor B9M can effectively improve cognition ability and significantly reduce Aβ plaques in APP/PS1 mice. And the optimal concentration of B9M is one in a thousand of that of Donepezil acted as the control treatment. The present data, together with our previous findings, suggest that B9M is a potential novel therapeutic strategy for AD by improving symptoms and slowing disease progression.
